# Genital self-amputation—its psychological urge

**DOI:** 10.1093/jscr/rjac569

**Published:** 2022-12-09

**Authors:** Srinath Ravichandran, Peter Mark Smith, Vincent Tang

**Affiliations:** Department of General Surgery, Stepping Hill Hospital, Stockport SK2 7JE, UK; Department of Urology, Stepping Hill Hospital, Stockport SK2 7JE, UK; Department of Urology, Stepping Hill Hospital, Stockport SK2 7JE, UK

## Abstract

Genital self-mutilation (GSM) is a rare phenomenon that can occur in patients with severe mental health illness. This case report highlights a rare case of self-inflicted bilateral testicular amputation and partial penile amputation in a patient who is a transwoman with a psychiatric history. The patient initially presented to urology in extremis with bilateral testicular amputation. The patient was resuscitated but required emergency surgery in the form of bilateral inguinal approach to ligate the cord and control haemostasis. The testes were not re-implanted as the patient refused and, after psychiatric discussion, was deemed to have capacity. She then re-presented within a week with self-inflicted partial amputation of penis. On both admissions, the patient had psychiatric evaluation but she was sectioned under the mental health act the second time. This case demonstrates how one can control haemostasis in the emergency scenarios of GSM and emphasizes the importance of psychiatric illness and evaluation in patients presenting with GSM.

## INTRODUCTION

Genital self-mutilation (GSM) in males is one of the rarest urological cases in the world. GSM occurs in various forms from simple laceration to complete bilateral testicular amputation and even penile amputation. It causes major functional and psychological consequences to the patient’s overall quality of life. Until now, just over a hundred of cases have been recorded in the literature dating back to the 20th century [1–4].

Although British Association of Urological Surgeons (BAUS) consensus statement suggests attempted re-implantation in centres with micro-vascular surgical skills and in those presenting within 12 hs, this is often not feasible due to patient being unstable, a lack of time for safe transfer and expertise [5].

## CASE REPORT

A Caucasian transwoman was referred from the Emergency Department to Urology with self-amputation of both the testicles. The patient had identified as a female for many years, undergone hormone manipulation and had a significant psychiatric background with episodes of severe depression, self-mutilation, alcohol abuse and suicidal ideation causing previous admissions under the mental health act.

The patient had previously seen the gender reassignment team, but they had refused immediate surgery on the basis of her alcohol intake and unstable mental health—instead arranging further review after a period of abstinence and stability. Despite abstinence and a period of stability for the prior 6 months, the patient did not see any other way out than GSM. She researched and obtained necessary tools (local anaesthetic, surgical scissors and a scalpel) from the internet in preparation and had been injecting local anaesthetic into her scrotum to ensure adequate numbing to allow a scalpel incision over the course of a few weeks.

On the day of this incident, the patient attended Emergency department (ED) stating her intention to perform GSM, where she was referred to crisis team for help but the patient did not choose to wait and left. She denied any thought of suicide and her intention was only to remove her genitals. She had approached ED multiple times even before for the same but never acted upon her intentions.

Later that day, after infiltrating local anaesthetic and using a scalpel bought online, she made an incision to her hemiscrotum to deliver the testicle that was then cut flush with the scalpel and removed. The process was then repeated on the contralateral side. As neither cord had been tied, she began to exsanguinate, at which point she phoned for emergency service help. The patient did not regret performing the act, it was not done with suicidal intent but after seeing the volume of blood, was concerned for her life.

In the hospital, the patient was resuscitated until haemodynamically stable in the ED, a compressive dressing was done and referred to the urology team. The patient received a stat dose of intravenous antibiotics, tranexamic acid and 1 unit of blood transfusion.

The patient was reviewed and observations were then stable. She stated she was very frustrated with waiting for her gender reassignment surgery and hence did it herself. The patient stated she would never want her testes back and if it was attempted, she would remove again. The risks and benefits of exploration, haemostasis and re-implantation of testes was thoroughly discussed. The patient understood this and expressed her wish for her life to be saved by controlling the bleeding but she did not consent to attempted re-implantation, nor did she want sperm cryo-preservation. She would not consent to surgery if we were to attempt re-implantation of her testes.

We had a telephone consultation with the on-call psychiatrist about the capacity of the patient. A key component to this decision was that she had not attempted to take her own life. The patient had ongoing severe bleeding that required urgent surgical intervention and was consenting to life-saving surgery but not attempted re-implantation. The psychiatric team agreed the patient had capacity and advised to proceed to surgery, without attempted re-implantation, and there was currently no need of sectioning .

The patient underwent scrotal exploration under general anaesthesia. On inspection, a grossly enlarged scrotum and two cuts were present with clots filled within the scrotum. The scrotal wounds were extended, clots evacuated and severe oedema of cord and connective tissues was seen with ongoing bleeding—causing difficulty in identifying the retracted cord. It was then decided to go through an inguinal approach in order to quickly control the bleeding: the cord was identified and double ligated at the deep inguinal ring, and the same was performed on the contralateral side. The scrotal wound was washed and closed in layers, leaving a corrugated drain.

During the hospital stay, the patient expressed wishes that she would perfrom self-amputation of her penis. She was then referred to the Crisis team who advised to restart her anti-psychotics and counselled her. The patient wanted to self-discharge on day 5 but the urology team felt that the patient was a risk to herself. Crisis team was informed about this decision and they advised the patient can be discharged as she had capacity to make this decision.

In 2 days’ time, the patient returned with attempted amputation of penis. The injuries were only superficial and she had de-gloved the penis without any other significant injury. The bleeding was settled and there was no threat to life. The patient initially refused any surgical intervention, but later gave consent for a repair. After being reviewed by the psychiatric team, she was sectioned under the mental health act for compulsory psychiatric evaluation.

## DISCUSSION

GSM is a rare phenomenon that mostly occurs within the background of severe mental health illness. The term Klingsor Syndrome has been suggested for GSM associated with religious delusions [6]. Klingsor was a magician who wanted to be accepted as a Knight of the Grail, a religious brotherhood. He castrated himself because of his inability to remain chaste to be accepted into this brotherhood [7, 8].

One of the issues with this patient is her waiting period for transgender surgical treatment - she felt that it was unfair, she could not wait any longer and being a transgender with the residual social stigma around the subject and everyday persecution felt by the patient, she felt like she had no way out other than to perform GSM.

Treatment of GSM necessitates a multidisciplinary approach, usually between the urologist, psychiatrist, psychologist and the primary care physicians. The main goal of surgical treatment includes life-saving measures as the imperative measurement, then restoration of anatomical continuity and function as much as possible.

In this scenario, one of the more challenging surgical aspects was control of haemostasis, hence the need for inguinal incisions. In terms of testicular genital mutilation not undergoing attempted re-implantation, if the cord is not visible due to oedema or retraction, then an inguinal approach may be necessary to quickly and safely control the cord pedicle and bleeding, as in this reported case.

Furthermore, although BAUS consensus statement recommends attempted re-implantation, in this case, the cord was retracted and oedematous, and without microsurgical expertise, this would be unlikely to be achievable [5]. Certainly, if re-implantation was to be attempted, it would have to be in a stable patient and life-saving treatment should not be delayed to allow the transfer to a centre with such expertise.

Though surgical treatment is very important, addressing mental health issue is another important modality of treatment to avoid readmission and further patient harm. Patients presenting acutely with GSM provide a challenge in assessing the capacity for surgical management and require strong MDT working and documentation to ensure they receive the correct care in a timely manner.

## CONCLUSION

GSM is an infrequent form of non-suicidal self-injury that occurs within a spectrum of severity. When dealing a case of GSM, clinicians should explore the motivations underlying the injury and consider a variety of psychiatric diagnoses along with surgical diagnosis. Even though the patient may not want re-implantation, it should be discussed and document thoroughly as the patient may not have capacity at that time of incident and capacity should always be formally assessed. Getting a psychiatric opinion before the procedure is important but should not delay any life saving surgery. The inguinal approach can be used in case of ongoing bleeding to control haemostasis quickly and safely. The patient should receive full psychiatric evaluation before discharged in order to prevent further episodes of GSM [9].
